# Perceived racial discrimination partially mediates racial‐ethnic disparities in dental utilization and oral health

**DOI:** 10.1111/jphd.12515

**Published:** 2022-06-21

**Authors:** Astha Singhal, John W. Jackson

**Affiliations:** ^1^ Health Policy and Health Services Research, Henry M. Goldman School of Dental Medicine Boston University Boston Massachusetts USA; ^2^ Epidemiology, Bloomberg School of Public Health John Hopkins University Baltimore Maryland USA

**Keywords:** access to care, health services research, disparity, racism, racial discrimination, tooth loss

## Abstract

**Objectives:**

Perceived racial discrimination has been associated with poor health outcomes, yet its impact on oral health disparities is not understood. We examine the role of perceived racial discrimination in healthcare settings in explaining racial‐ethnic disparities in dental visits and tooth loss.

**Methods:**

We used behavioral risk factor surveillance system (BRFSS) data for 2014 from four diverse states (AZ, MN, MS and NM) that included “reactions to race” module. Using Poisson regression to estimate risk ratios, we employed inverse odds ratio(IOR)‐weighted estimation for mediation analyses to estimate the role of perceived discrimination, after equalizing other confounders and risk factors.

**Results:**

We found that among those with similar risk factors, those who experienced racial discrimination were 15% less likely to visit a dentist, and 12% more likely to have tooth loss than those who were treated same as other races. Both Hispanics and non‐Hispanic Blacks were 26% less likely to visit a dentist (for Hispanics, RR = 0.74, 95%CI: 0.69–0.78; for non‐Hispanic Blacks, RR = 0.74, 95%CI: 0.70–0.79), and non‐Hispanic Blacks were 36% more likely to have tooth loss relative to non‐Hispanic Whites with similar risk factors (RR = 1.36, 95%CI: 1.28–1.45). Perceived discrimination appears to contribute to racial‐ethnic disparities in dental utilization among Hispanics, and disparities in tooth loss among non‐Hispanic Blacks, relative to non‐Hispanic Whites.

**Conclusions:**

Perceived racial discrimination partially explains the racial‐ethnic disparities in dental utilization and tooth loss among those who otherwise share the same risk factors for these outcomes. Addressing discrimination and systemic racism can reduce the racial‐ethnic disparities in oral health.

## INTRODUCTION

Oral health is an integral component of overall health and has a significant impact on an individual's well‐being and productivity [1]. Despite that, several barriers exist to accessing oral health care, and dental care utilization has been declining among non‐elderly adults in the US [2]. Regular dental visits can facilitate prevention, early diagnoses and treatment of oral diseases and result in improved clinical outcomes and quality of life [3]. Untreated dental disease could lead to serious complications such as tooth loss, systemic infections and can sometimes be fatal. Populations that are unable to access the dental care delivery system turn to hospital emergency rooms, that add to the societal costs yet provide no definitive dental treatment [4]. The global economic impact of dental diseases has been estimated at US$442 billion in 2010 [1].

One of the adverse outcomes that can be reduced through regular dental visits is tooth loss [3]. Tooth loss is associated with poorer oral health, nutrition, systemic health and quality of life [5, 6]. Tooth loss can have additional economic impact such as reducing the likelihood of finding employment [7]. While the prevalence of tooth loss has been declining over the past decades [5], not all population groups have experienced equal gains [8]. National estimates suggest that the gains in tooth retention were concentrated among non‐poor older adults, and non‐Hispanic Blacks continued to experience lower tooth retention [8].

Although some studies suggest that racial disparities in children's oral health and dental care utilization have declined over the past 50 years [9], disparities have persisted among minority adults. Several studies report that racial‐ethnic minorities, particularly Blacks, experience disproportionately more oral health problems and these disparities increase with age [10, 11]. Studies among older Americans found that not only Black Americans have greater number of decayed and missing teeth on average, but the disparities in missing teeth have increased between Black and White Americans [11]. On the other hand, Whites consistently have greater number of filled teeth on average, suggesting better access to comprehensive dental care [11].

Racial‐ethnic disparities have persisted despite research, programmatic and policy interventions aimed to eliminate them. Several factors contribute to racial‐ethnic disparities, such as social, demographic, economic, cultural and environmental factors. One aspect of the environment that may contribute to racial‐ethnic disparities but has not been examined in the context of oral health disparities is perceived racial discrimination in healthcare settings [12].

Racial discrimination has been defined as differential treatment on the basis of race, or treatment on the basis of inadequately justified factors other than race, that disadvantages a racial group [13]. This definition is closely related to the notion of interpersonal racism which involves differential actions towards others according to their race, which may be intentional or unintentional and involving acts of commission and omission and manifest as lack of respect, suspicion, scapegoating, and dehumanization [14]. From medical literature, we know that perceived racial discrimination leads to distrust in the health care system, stress‐induced depression, poorer health behaviors and poor health outcomes [15, 16]. When examining the dental delivery system and oral health outcomes, however, the evidence is limited and mixed. For example, one study in Brazil reported that racial disparities in tooth loss are partially explained by socio‐demographic differences, but not by behavior and self‐reported discrimination [17]. Yet, other studies among aboriginal populations in Australia and Canada reported that self‐reported racism was a barrier to accessing dental care and associated with higher prevalence of toothache [18, 19]. A recent study in the US reported that perceived discrimination is not associated with dental utilization, but the emotional impact of discrimination, such as feeling angry or sad due to how one was treated based on their race, was associated. [20] However, no study in the U.S. thus far has examined if perceived racial discrimination mediates racial‐ethnic disparities in oral health. This is important because racial‐ethnic disparities have persevered despite past initiatives, and addressing racial discrimination in healthcare settings could be key to eliminating these oral health disparities.

To our knowledge, this is the first U.S. study to examine the potential role of perceived racial discrimination in healthcare settings in explaining racial‐ethnic disparities in dental utilization and tooth loss. Using representative population from four states, we examine the role of perceived racial discrimination on dental utilization and tooth loss among Hispanics, non‐Hispanic Blacks relative to non‐Hispanic Whites. We hypothesize that those who perceive being discriminated based on their race while seeking healthcare will be less likely to engage in preventive behaviors, such as regular dental visits, and hence will be more likely to have adverse health outcomes, such as tooth loss. Given that racial‐ethnic minorities experience discrimination at a greater rate [21], we hypothesize that perceived racial discrimination will, in a statistical sense, explain some of the racial‐ethnic disparities in oral health outcomes.

## STUDY DATA AND METHODS

### Data source and study design

We used data from the behavioral risk factor surveillance system (BRFSS), which is a nationally representative annual survey that collects information on self‐reported preventive health behaviors and risk behaviors. It is a telephone‐based survey that is conducted at the state level, using random, population‐based samples of non‐institutionalized adults. The BRFSS data is publicly available for all 50 states and Washington DC. We used data for 2014, which was the latest year available that included “reactions to race” module that measures perceived discrimination in healthcare settings. Our sample comprised of data from all four states (AZ, MN, MS and NM) that included the optional “reactions to race” module in their 2014 survey.

### Study population and variables

Our study population included all adults aged 18 or more who self‐identified as either Hispanic, non‐Hispanic White or non‐Hispanic Black, and residing in one of the four states‐AZ, MN, MS and NM.

We had two main *dependent variables*‐an indicator of having visited a dentist in the past 12 months, and an indicator of having lost one or more permanent teeth due to dental disease. Dental visit in past 12 months is an accepted measure of utilization and access to dental care, and is the only dental measure included in healthcare effectiveness data and information set (HEDIS) by the National Committee for Quality Assurance [22]. Loss of permanent teeth is an adverse outcome that indicates poor oral health status [6].

As we were interested in examining racial‐ethnic disparities, our main *independent variable* was race‐ethnicity. This variable was categorized into three classes‐Hispanic, non‐Hispanic Black and non‐Hispanic White. The non‐Hispanic others group was excluded from our analyses as it was a heterogeneous group of various race‐ethnicities making it difficult to interpret the results and generalize its implications.

The *mediator* that we were interested in examining was derived from the optional “reactions to race” module's survey item. We were specifically interested in perceived racial discrimination in healthcare settings, hence we derived our measure from the following question as a categorical variable:

In the past 12 months when seeking health care, do you feel your experiences were worse than, the same as, or better than for people of other races?

The “reactions to race” module used in BRFSS has been validated and used in several studies that have examined racial discrimination [23, 24, 25] though, conceptually, it may be viewed as related but somewhat distinct from more traditional definitions and measures of discrimination. Frequently used assessments of perceived discrimination ask respondents about unfair treatment that is explicitly attributed to race in specific settings (school, employment, housing, medical care, store/restaurant), or summarize unfair treatment attributed to race across several routine life settings (work, police, education, housing, bank, receipt of services) [26, 27]. The BRFSS item shares the setting‐specific frame of medical care but does away with assessments of perceived intent of the physician, care team, or healthcare institution. In its place the item asks respondents to assess whether ultimately, regardless of mechanism, their care‐related experiences nonetheless differed by race. In this sense it may capture intentional discrimination and implicit bias but it is more broad than this. Ostensibly, it could also pick up ways by which experiences of care vary by race indirectly through a provider or institution's practices, policies, and structures. In this sense the item may be viewed as possibly picking up effects of racist policies or practices, which have been recently defined by some as policies and practices that produce adverse outcomes for racially marginalized groups, regardless of the policies and practices' intent [28]. It is aligned with the definition of discrimination provided earlier. That definition allows for differential treatment by race to occur directly when race or indirectly when inappropriate factors associated with race (such as socioeconomic status or position) are used to guide care decisions [13]. Finally, and perhaps most interestingly, the item is in some sense more global than the individual‐specific experience of unfair treatment. A White person believing that their own experience of care was on average better than the experience of a Black person would be consistent with the notion of a racist practice or policy in the antiracist sense, and the BRFSS measure would capture this [28]. Thus, stepping back, the BRFSS item may capture many of the nuanced forms in which experiences of care may vary by race, which may include intentional forms of discrimination, including interpersonal racism, as well as non‐intentional forms that play out in institutional practices and policies, which some have referred to as structural racism [29].

To address potential confounding, our analyses accounted for other *covariates* and possible confounders that included: household income, educational attainment, age, sex, marital status, residential location, home ownership, employment status, health insurance, type of health insurance, self‐reported general health status and smoking status. These variables were carefully chosen based on their relationship with the outcomes, predictor, and mediator. If a variable was associated with either outcome in bivariate analyses, it was included in the multivariable models we describe next.

### Analysis

Using Poisson regression to obtain risk ratios, we examined the relative racial/ethnic disparity in the risk of having a dental visit, and also loss of permanent teeth, both marginally and within levels of the confounders.

Next, to explore the effect of racial discrimination on racial‐ethnic disparities in dental visit and tooth loss among those with the same values for confounders, we used inverse odds ratio(IOR)‐weighted estimation for mediation analyses [30]. For the Hispanic group, we computed weights for each observation that were equal to the ratio of (i) the probability of being non‐Hispanic White (reference group) given the specific values of discrimination and the covariates [numerator] (ii) the probability of being Hispanic given the specific values of discrimination and covariates [denominator]. We also computed weights in this fashion for non‐Hispanic Blacks. This allowed for variation across the four levels of racial discrimination, and also their effects, while making the distribution similar across racial‐ethnic groups with same level of confounders. To account for potentially different patterns of discrimination (and different factors that may affect discrimination patterns) in the four states included in our analyses, we included state and state × discrimination interaction in estimating our weights. Then, we computed final weights as a product of the computed IOR‐weights and design weights made available with BRFSS data. Finally, weighted multivariate Poisson regressions were conducted to obtain risk ratios indicating racial/ethnic disparity in dental visit (and tooth loss) after equalizing the conditional distribution of discrimination in healthcare settings. Using the inverse odds ratio weighting approach allowed us to hold similar the patterns of discrimination across racial groups to assess its association with disparities while accounting for potential confounders and heterogeneous effects of discrimination, something that could not have been done using the traditional Barron and Kenney approaches [30, 31]. With the exception of the weight estimation, all of the analyses presented in the manuscript accounted for the complex survey design.

## RESULTS

**FIGURE 1:**
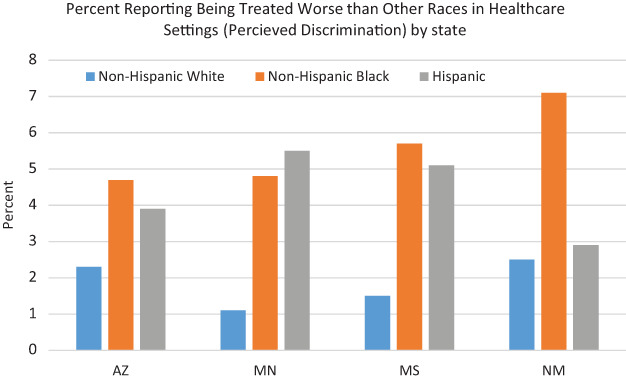
Distribution of perceived racial discrimination across racial‐ethnic groups by state [Color figure can be viewed at wileyonlinelibrary.com]

The sample consisted of 36,790 adults, representing 13.1 million residents across the four states. We excluded “non‐Hispanic other” group due to its heterogeneous nature, so our final sample size was 33,924 representing about 12 million adult residents. About 38% of the weighted sample were residents of Arizona, 32% were from Minnesota, 18% from Mississippi, and 12% from New Mexico. Figure 1 shows the distribution of perceived racial discrimination in healthcare settings by various racial‐ethnic groups in each study state. While there are different patterns that reflect the unique social history and culture of racism in individual states, overall the minorities were much more likely to report being discriminated against. We present pooled results from here on that include state‐fixed effects and state × discrimination interaction in estimating our weights to account for inherent differences and varying patterns of discrimination between individual states.

**FIGURE 2:**
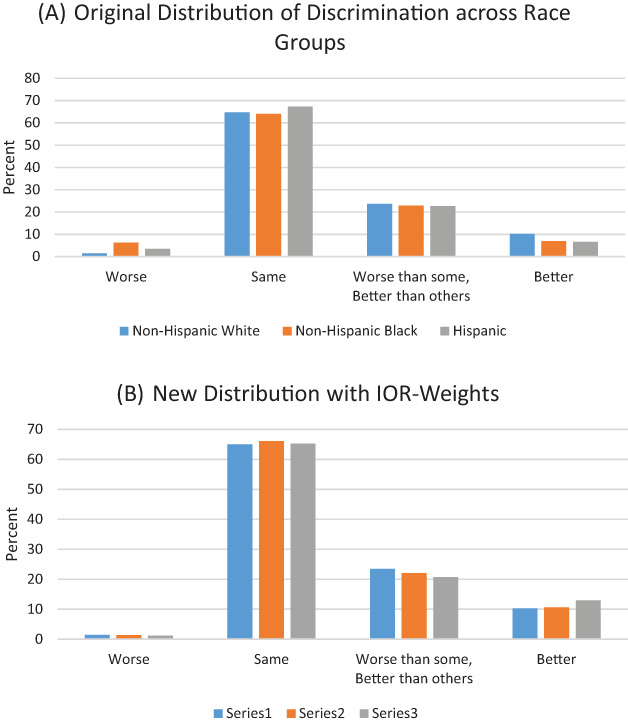
Distribution of perceived racial discrimination across racial‐ethnic groups before and after inverse odds ratio weighting [Color figure can be viewed at wileyonlinelibrary.com]

Figure 2 shows the distribution of racial discrimination across racial‐ethnic groups in the original sample (1a) and transformed distribution after applying the IOR‐weights (1b). When asked about how they were treated based on their race relative to other races when seeking healthcare in the past year, 6.2% of non‐Hispanic Blacks reported being treated worse, followed by 3.5% of Hispanics and only 1.4% of non‐Hispanic Whites. In contrast, 10.2% of non‐Hispanic Whites reported being treated better than other races, relative to only 6.9% of non‐Hispanic Blacks and 6.6% of Hispanics. However, after applying the IOR‐weights (1b), the distribution of discrimination patterns became more uniform across racial‐ethnic groups, hence achieving the intended purpose of statistically equalizing discrimination distribution across racial‐ethnic groups.

The sample distribution and the crude risk ratios for annual dental visit and any permanent tooth loss are presented in Table 1. Our sample included 74% non‐Hispanic White, 13.7% non‐Hispanic Black and 12.3% Hispanic adults. Overall, about 66% adults reported visiting a dentist in the past year and 44% reported having lost at least one permanent tooth.

**TABLE 1:** Population characteristics and crude risk ratios for annual dental visit and tooth loss

	Population characteristics	Dentist visit in the past year		Any permanent tooth loss	
	%	RR	95% CI	RR	95% CI
Race‐ethnicity^a^					
Non‐Hispanic White	74.0	1		1	
Non‐Hispanic Black	13.7	**0.74**	**0.70–0.79**	**1.36**	**1.28–1.45**
Hispanic	12.3	**0.74**	**0.69–0.78**	0.96	0.89–1.03
Treatment based on race in healthcare settings					
Worse than others	2.2	**0.63**	**0.55–0.74**	**1.44**	**1.29–1.61**
Same as others	67.9	1		1	
Better than others	7.6	1.05	1.00–1.11	1.07	0.99–1.15
Worse than some, better than others	22.4	**0.92**	**0.89–0.96**	**1.13**	**1.08–1.19**
Annual household income					
< $25,000	25.8	**0.54**	**0.51–0.56**	**1.79**	**1.70–1.89**
$25,000–$49,999	22.4	**0.76**	**0.74–0.79**	**1.48**	**1.39–1.56**
$50,000+	38.2	1		1	
Do not know/refused	13.6	**0.77**	**0.74–0.81**	**1.39**	**1.29–1.49**
Education					
High school or less	41.4	**0.81**	**0.79–0.83**	**1.36**	**1.32–1.39**
Attended college/technical school	33.8	1.01	0.99–1.03	1.01	0.98–1.04
Graduated from college/technical school	24.8	1		1	
Age					
18–24 years	12.4	1		1	
25–34 years	16.9	**0.89**	**0.80–0.94**	**2.22**	**1.78–2.77**
35–44 years	15.8	0.98	0.92–1.05	**2.76**	**2.24–3.41**
45–54 years	17.7	0.99	0.93–1.05	**3.72**	**3.03–4.56**
55–64 years	17.4	1.03	0.97–1.10	**4.5**	**3/68–5.51**
65 + years	19.9	1.03	0.97–1.09	**5.34**	**4.37–6.52**
Sex					
Male	48.5	1		1	
Female	51.5	**1.04**	**1.02–1.05**	1.01	0.98–1.03
Marital status					
Married	52.5	1		1	
Not married (never married, divorced, widowed, separated, other)	47.5	**0.89**	**0.88–0.91**	0.98	0.96–1.01
Live in a MSA					
Yes	25.4	**0.91**	**0.90–0.92**	0.98	0.96–0.99
No	74.6	1		1	
Home owner					
Yes	72.7	1		1	
No	27.3	**0.84**	**0.83–0.86**	1	0.98–1.03
Employment					
Wages or self‐employed	58.4	1		1	
Retired	17.7	**1.03**	**1.00–1.06**	**1.83**	**1.76–1.91**
Others (unemployed, student, homemaker, unable to work)	23.8	**0.81**	**0.78–0.85**	**1.29**	**1.22–1.37**
Health insurance					
Yes	87.6	1		1	
No	12.4	**0.73**	**0.70–0.77**	1.03	0.99–1.07
Type of insurance					
Private insurance	55.0	1		1	
Medicare	16.7	**0.84**	**0.81–0.87**	**1.97**	**1.88–2.05**
Medicaid/state plan	7.1	**0.64**	**0.59–0.69**	**1.61**	**1.50–1.74**
Others (tricare, other source, none)	21.2	**0.64**	**0.60–0.67**	**1.31**	**1.23–1.39**
General health status					
Excellent/very good/good	83.3	1		**1**	
Fair/poor	16.7	**0.84**	**0.82–0.86**	**1.32**	**1.29–1.35**
Smoker					
Current	18.1	**0.7**	**0.66–0.74**	**1.65**	**1.56–1.74**
Former	24.5	0.98	0.95–1.01	**1.59**	**1.52–1.66**
Never	57.5	1		1	
Visited a dentist in the past year	65.7	—	—	—	—
Lost some permanent tooth	44.2	—	—	—	—

*Note*: Bolded values indicate statistical significance determined at *p* value < 0.05.

Total percentage may add up to more than 100 due to rounding.

^a^ Non‐Hispanic other group was excluded from our analyses.

Table 2 shows the crude and adjusted risk ratios for annual dental visit and tooth loss across levels of racial discrimination. We found that those who reported being treated worse than other races were 37% less likely to have a dental visit (RR = 0.63, 95% CI: 0.55–0.74) than those who reported being treated same as other races, and this difference reduced to 15% (RR = 0.85, 95% CI: 0.73–0.98) after adjusting for race and other confounders. On the other hand, those who were treated worse had 44% greater risk of tooth loss (RR = 1.44, 95% CI: 1.28–1.61) than those who were treated same as other races, which declined to 12% greater risk (RR = 1.12, 95% CI: 1.02–1.24) after adjusting for race and other confounders.

**TABLE 2:** Crude and adjusted risk ratios for annual dental visit and tooth loss across discrimination categories

Treatment based on your race in healthcare settings	Dentist visit in the past year			Any permanent tooth loss		
	Crude risk ratios					
	RR	95%CI	*p* value	RR	95%CI	*p* value
Worse	**0.63**	**0.55–0.74**	**<0.0001**	**1.44**	**1.28–1.61**	**<0.0001**
Same	1			1		
Better	1.05	1.00–1.11	0.0518	1.07	0.99–1.16	0.0771
Worse than some, better than others	**0.92**	**0.89–0.96**	**<0.0001**	**1.13**	**1.08–1.19**	**<0.0001**

	Adjusted risk ratios					
	RR	95%CI	*p* value	RR	95%CI	*p* value
Worse	**0.85**	**0.73–0.98**	**0.0228**	**1.12**	**1.02–1.24**	**0.0234**
Same	1			1		
Better	1.02	0.97–1.07	0.3941	0.98	0.91–1.05	0.5343
Worse than some, better than others	0.97	0.94–1.00	0.0804	1.02	0.97–1.06	0.4886

*Note*: Adjusted for race‐ethnicity, age, sex, marital status, income, education, rural/urban residence, employment status, health insurance, type of health insurance, home ownership, general health and smoking status. Bold values were statistically significant with *p*‐value < 0.05.

Results for the crude disparity, the disparity after adjusting for confounders, and the residual disparity after further equalizing discrimination across racial‐ethnic groups are reported in Table 3. The crude disparity was 26% lower likelihood of annual dental visit among both Hispanics (RR = 0.74, 95% CI: 0.70–0.79) and non‐Hispanic Blacks (RR = 0.74, 95% CI: 0.69–0.78) relative to non‐Hispanic Whites. The disparity was much smaller (7%) among those with the same levels of confounders. Further, accounting for discrimination made the differences not significant across both racial‐ethnic minority groups, but reduced the absolute disparity from 7% to 1% only among Hispanics, relative to non‐Hispanic Whites.

**TABLE 3:** Crude and conditional disparities in annual dental visit and tooth loss across racial‐ethnic groups, before and after equalizing discrimination via IOR‐weighting

Race‐ethnicity	Dentist visit in the past year			Any permanent tooth loss		
	Crude risk ratios					
	RR	95%CI	*p* value	RR	95%CI	*p* value
Non‐Hispanic White	1			1		
Non‐Hispanic Black	**0.74**	0.70–0.79	**<0.0001**	**1.36**	1.28–1.44	**<0.0001**
Hispanic	**0.74**	0.69–0.78	**<0.0001**	0.96	0.89–1.03	0.2252

	Adjusted risk ratios before equalizing discrimination					
	RR	95%CI	*p* value	RR	95%CI	*p* value
Non‐Hispanic White	1			1		
Non‐Hispanic Black	**0.93**	0.87–0.99	**0.0323**	**1.32**	1.25–1.39	**<0.0001**
Hispanic	**0.93**	0.87–0.98	**0.0080**	0.97	0.91–1.04	0.4425

	Adjusted risk ratios after equalizing discrimination					
	RR	95%CI	*p* value	RR	95%CI	*p* value
Non‐Hispanic White	1			1		
Non‐Hispanic Black	0.92	0.83–1.02	0.1172	**1.15**	1.03–1.28	**0.0162**
Hispanic	0.99	0.92–1.06	0.7955	0.99	0.88–1.11	0.8465

*Note*: Adjusted for race‐ethnicity, age, sex, marital status, income, education, rural/urban residence, employment status, health insurance, type of health insurance, home ownership, general health and smoking status.

“Equalizing discrimination” refers to the use of Inverse Odds Ratio (IOR) weights to create an equal distribution of discrimination across racial‐ethnic groups to determine how that affects the relationship between race‐ethnicity and dental outcomes.

Similarly, when examining permanent tooth loss, there was a 36% greater crude risk of tooth loss among non‐Hispanic Blacks (RR = 1.36, 95% CI: 1.28–1.44) relative to non‐Hispanic Whites. Among those with the same values for confounders, the disparity was 32% (RR = 1.32, 95% CI: 1.25–1.39). This disparity further reduced to 15% after equalizing discrimination (RR = 1.15, 95% CI: 1.03–1.28), but remained statistically significant. The reduction in absolute disparity from 32% to 15% indicates that perceived discrimination impacts tooth loss among non‐Hispanic Blacks compared to non‐Hispanic Whites. There were no significant differences in risk of tooth loss between Hispanics and non‐Hispanic Whites.

## DISCUSSION

Our results confirm that substantial racial‐ethnic disparities exist in dental utilization and tooth loss. Both Hispanics and non‐Hispanic Blacks were less likely to visit a dentist in the past year, and non‐Hispanic Blacks more likely to experience loss of permanent teeth relative to non‐Hispanic Whites. While socio‐demographics and other confounders may explain some of these disparities, significant disparities persist and have been resistant to past policy and programmatic interventions [9, 11, 12]. We examined the role of perceived racial discrimination in healthcare settings and found that discrimination partially explains racial‐ethnic disparities in dental utilization and tooth loss among those with otherwise similar risk factors for these oral health outcomes. Our results indicate that perceived racial discrimination contributes to racial‐ethnic disparities in dental utilization among Hispanics, and to disparities in tooth loss among non‐Hispanic Blacks, relative to non‐Hispanic Whites. Specifically, perceived racial discrimination accounts for about 6.5% of this conditional disparity among those with the same confounders in dental utilization between Hispanics and non‐Hispanic Whites. Among non‐Hispanic Blacks, discrimination did not reduce the disparity in dental utilization, but it accounts for about 12.9% of the conditional disparity in tooth loss compared to non‐Hispanic Whites. Establishing the causality of these estimates will require further study with longitudinal data. However, it is interesting that discrimination seems to explain low dental visits among Hispanics but not Blacks, while it seems to explain the disparity in tooth loss among Blacks. Why perceived discrimination has such differing effect on disparities in oral health outcomes of these two racial‐ethnic groups is not entirely clear.

While the literature on racial discrimination on oral health outcomes is sparse, examination of general health outcomes reveals that experiencing discrimination can trigger stress neurobiology and result in poor health behaviors, increased likelihood of substance abuse and poor physical and mental health [32]. Our results concur with these studies as we found that those who experienced racial discrimination in healthcare settings were significantly less likely to visit a dentist in past year and more likely to report loss of permanent teeth, even after holding fixed the risk factors for tooth loss and predictors of utilization. While neurobiological events triggered by discrimination may explain the outcomes, a more proximal explanation is that those who experience discrimination when seeking healthcare are more likely to distrust the healthcare system, and avoid it leading to lower utilization and poorer health outcomes [16].

Our results indicate that for utilization, the disparity was much smaller among those who shared the same predictors (by 26%), whereas for tooth loss the disparity was only slightly smaller (by 1%–3%). This can be attributed to the fact that BRFSS data includes several variables that determine access to dental care, including socio‐demographics, economic constraints and insurance status. However, tooth loss is a more complex outcome because even though it is an adverse outcome, tooth loss can partially reflect past access to dental care as visiting a dentist for extraction is a pre‐requisite in most cases of tooth loss. Although, we do account for known confounders of tooth loss, (including age, general health and smoking status) several other factors such as chronic diseases, medications and gum disease could lead to tooth loss, information on which were not available in the data.

Racial discrimination in healthcare settings could be a result of both explicit and implicit bias on the part of the provider team. Explicit bias, or racism, is deeply rooted in the historical aspects of our society. Systemic racism pervades the structures, practices and policies of social institutions, and has a detrimental effect on several aspects of human lives, including health [21, 33]. Cultural bias could lead to implicit bias that operates through negative stereotypes that are supported by long‐standing systemic oppression and racist policies. Providers' implicit bias against racial‐ethnic minorities has been associated with differences in diagnoses, clinical assessment of pain, treatment decisions and patients' overall healthcare experience [34, 35]. When clinical information is limited, clinicians may rely on implicit biases about the social groups to which patients belong [36]. For example, if Blacks have lower socioeconomic status on average, and those with low socioeconomic status are stereotyped as non‐adherent, a provider may stereotype a Black person with low socioeconomic status as potentially non‐adherent (and thus steer the patient away from treatments that work best with high levels of adherence) if the provider does not know much about the patients' clinical history or level of activation. Such uncertainty can occur when patient‐provider communication is poor, which may be more likely when there is race, ethnicity, or language‐discordance between providers and patients [37]. There exists some evidence that concordant relationships in dentistry are associated with greater satisfaction and communication [38]. However, dentistry has historically been a non‐Hispanic white male profession [39]. A survey of national sample of dental practices shows that patients prefer race and language concordant providers with 45% of Hispanic dentists' patient being Hispanic, compared to only 8.5% of non‐Hispanic white dentists' [39]. Similarly, 62% of black dentists' patients were black, while only 10.5% of white dentists' patient being black [39]. While the diversity of dental profession is improving, only 6% of professionally active dentists in the US are underrepresented minorities [39]. Recruiting and training more providers from disadvantaged minority backgrounds can improve the racial imbalance between patient and provider populations, such as the dental pipeline program [40].

Health care providers, including oral health practitioners, need to be cognizant of the ways in which disparate care can arise in healthcare settings, and the contribution of their own biases to it. Dental professionals must be provided with the tools and training to address these barriers, such as adequate and appropriate cultural competency training in dental schools [41], required continuing education on implicit bias and training to reduce such biases [42]. While trainings by themselves may not be effective, when supported by other interventions to address institutional racism, such as diversity and inclusion initiatives, has the potential to begin to dismantle both implicit and explicit bias [43, 44]. It is imperative that future efforts to eliminate racial‐ethnic disparities consider including discrimination and other dimensions of racism, especially when seeking healthcare. Conceptual models linking individual level and structural factors to disparate dental outcomes need to be developed and tested, to inform interventions that target the underlying mechanisms of oral health disparities.

### Limitations

There were some limitations to our study. First, since our data are cross‐sectional, it limits our ability to make any causal inferences from the mediation analyses. We did adjust for several risk factors of tooth loss and predictors of utilization that may confound their relationships with discrimination. Yet, the temporality between some covariates and the dependent variables is unclear. Future research should confirm our results with temporally ordered data. Second, while we specifically examined the effect of racial discrimination in healthcare settings, we could not account for the timing, patterns, and severity of such discriminatory experience, which could have different impacts on oral health outcomes. Moreover, same levels of discrimination may have different implications across racial groups [45]. Third, only four states included the “reactions to race” module and hence could be included in our study. This limits our ability to generalize these results to other states that were not included in the study. Finally, information on important confounders for dental visit (such as dental insurance) and tooth loss (such as systemic diseases, medication use, etc.) were not available in the BRFSS data.

## CONCLUSIONS

Our study provides evidence that there are significant disparities in dental care utilization and tooth loss between racial‐ethnic groups, even among those with shared socio‐demographic and other risk factors. We found that racial discrimination in healthcare settings may be contributing to these disparities, with those treated worse than other races being less likely to visit a dentist and more likely to lose their teeth. These findings indicate that future research, interventions and policies should examine race in a wider socio‐historical context, and address racial discrimination to reduce persistent racial‐ethnic disparities in oral health.
